# Prognostic value of preoperative dynamic contrast-enhanced MRI perfusion parameters for high-grade glioma patients

**DOI:** 10.1007/s00234-016-1741-7

**Published:** 2016-10-29

**Authors:** Agne Ulyte, Vasileios K. Katsaros, Evangelia Liouta, Georgios Stranjalis, Christos Boskos, Nickolas Papanikolaou, Jurgita Usinskiene, Sotirios Bisdas

**Affiliations:** 1 Faculty of Medicine, Vilnius University, Vilnius, Lithuania; 2 Department of Advanced Imaging Modalities – CT and MRI, General Anticancer and Oncological Hospital “St. Savvas”, Athens, Greece; 3 Department of Neurosurgery, Evangelismos Hospital, University of Athens, Athens, Greece; 4 Department of Radiation Oncology, General Anticancer and Oncological Hospital “St. Savvas”, Athens, Greece; 5 Department of Radiology, Centre for the Unknown, Champalimaud Foundation, Lisbon, Portugal; 6 National Cancer Institute, Vilnius, Lithuania; 7 Affidea Lietuva, Vilnius, Lithuania; 8 Department of Neuroradiology, The National Hospital for Neurology and Neurosurgery, University College London Hospitals, Box 65, Queen Square 8-11, London, WC1N 3BG UK

**Keywords:** Dynamic contrast-enhanced MRI, Gliomas, Perfusion transfer coefficient, Vascular plasma volume fraction

## Abstract

**Introduction:**

The prognostic value of the dynamic contrast-enhanced (DCE) MRI perfusion and its histogram analysis-derived metrics is not well established for high-grade glioma (HGG) patients. The aim of this prospective study was to investigate DCE perfusion transfer coefficient (K^trans^), vascular plasma volume fraction (v_p_), extracellular volume fraction (v_e_), reverse transfer constant (k_ep_), and initial area under gadolinium concentration time curve (IAUGC) as predictors of progression-free (PFS) and overall survival (OS) in HGG patients.

**Methods:**

Sixty-nine patients with suspected anaplastic astrocytoma or glioblastoma underwent preoperative DCE-MRI scans. DCE perfusion whole tumor region histogram parameters, clinical details, and PFS and OS data were obtained. Univariate, multivariate, and Kaplan–Meier survival analyses were conducted. Receiver operating characteristic (ROC) curve analysis was employed to identify perfusion parameters with the best differentiation performance.

**Results:**

On univariate analysis, v_e_ and skewness of v_p_ had significant negative impacts, while k_ep_ had significant positive impact on OS (*P* < 0.05). v_e_ was also a negative predictor of PFS (*P* < 0.05). Patients with lower v_e_ and IAUGC had longer median PFS and OS on Kaplan–Meier analysis (*P* < 0.05). K^trans^ and v_e_ could also differentiate grade III from IV gliomas (area under the curve 0.819 and 0.791, respectively).

**Conclusions:**

High v_e_ is a consistent predictor of worse PFS and OS in HGG glioma patients. v_p_ skewness and k_ep_ are also predictive for OS. K^trans^ and v_e_ demonstrated the best diagnostic performance for differentiating grade III from IV gliomas.

## Introduction

High-grade gliomas (HGGs) comprise a group of aggressive primary brain tumors with a heterogeneous prognosis: median survival time is around 1 year for glioblastoma [1], but 8 % of patients survive up to 2.5 years or longer [2]. Until recently, WHO glioma grade has been considered the most robust prognostic factor, but this view is challenged by HGG genetic and imaging research [3–6]. Perfusion parameters, such as relative cerebral blood volume (rCBV), are among the most consistently recognized independent predictors of survival [4, 7]. In line with the working hypothesis, perfusion parameters correlate with tumor vascularity and properties of vessels, which, in turn, are strongly associated with glioma grade progression and poorer survival [8, 9]. Therefore, perfusion is viewed as a powerful prognostic tool to stratify gliomas according to their aggressiveness, predict patient survival, and eventually assist in treatment choice.

The most widely used MRI techniques to measure perfusion include dynamic susceptibility contrast-enhanced (DSC) and dynamic contrast-enhanced (DCE) perfusion. These two techniques have considerable differences. Firstly, DSC perfusion is based on T2* (susceptibility-weighted) signal change in the presence of contrast material, while DCE perfusion is calculated from T1 signal change due to contrast enhancement. Secondly, they yield different perfusion parameters. DSC primarily measures cerebral blood volume (CBV) and is therefore concerned with quantity of vascularization. In contrast, DCE perfusion describes the quality of vessels and quantifies parameters related to contrast extravasation—vascular permeability (leakiness). Increased blood vessel permeability is a cardinal feature of neoangiogenesis and is reflected in increased contrast transfer rate, K^trans^. DCE perfusion also enables calculation of other parameters such as extracellular volume fraction (v_e_), reverse transfer rate (k_ep_), vascular plasma volume fraction (v_p_), and initial area under the gadolinium concentration time curve (IAUGC). These parameters provide additional information about physiological tumor properties and could afford independent insight about clinical tumor behavior, especially as DCE perfusion parameters were shown not to correlate with DSC [10] or diffusion parameters [11] closely. DCE perfusion parameters have already been demonstrated to be diagnostic for glioma grade [12–17] and correlate with several proxies of hypoxia like microvascular density [18, 19] and HIF-1α and vascular endothelial growth factor (VEGF) expression [20]. DCE perfusion also has predictive value for treatment with neoangiogenesis inhibitors [21, 22] and chemoradiotherapy [23].

Thus far, research on the prognostic value of DCE perfusion for HGG patient survival has been scarce—only a few studies have been conducted [10, 20, 24–26], some of them presenting contradicting results [27], focusing mostly on K^trans^ and v_p_, and some of them employing the hot-spot method, where only tumor regions with subjectively highest parameter values are analyzed. We set out to partially fill this knowledge gap with a larger study sample and a comprehensive whole tumor volume histogram analysis of more DCE-MRI pharmacokinetic modeling parameters. The aim of this study was to investigate DCE perfusion parameters (K^trans^, v_p_, v_e_, k_ep_, and IAUGC) as independent predictors of progression-free survival (PFS) and overall survival (OS) in HGG patients. Secondary objectives were to examine DCE performance to differentiate glioma grade III from IV and other histological characteristics of gliomas.

## Materials and methods

### Patient population

This prospective study was approved by the Institutional Review Board, and permission was granted for the use of images and medical records. The study was compliant with the declaration of Helsinki. All relevant information about the examination and the study was thoroughly explained for the patients, and informed consent was obtained.

From October 2009 to March 2015, 69 patients (41 men, 28 women; median age 55 years; range 21–77 years) with conventional or spectroscopic MRI findings suggestive for a primary high-grade glioma (either anaplastic astrocytoma or glioblastoma (GBM)) were enrolled in the study. Exclusion criteria were any prior brain tumors, tumor histology other than GBM or anaplastic astrocytoma, and lack of informed consent. All patients received DCE-MRI prior to maximal surgical resection to confirm tumor histology. O^6^-methylguanine-DNA methyltransferase (MGMT) promoter methylation status, isocitrate dehydrogenase (IDH) 1 and 2 mutation, and Ki67 immunostaining index (MIB-1) were also investigated. Clinical variables, such as age, sex, Karnofsky performance score, and ensuing treatment, were recorded. All patients were operated, and the majority also received adjuvant treatment—radiotherapy, chemotherapy, or combined therapy. Patients were treated according to the current ESMO Clinical Practice Guidelines [28–30] and were regularly followed up, including MR imaging post-operatively, 3 weeks, 2, 3, 6, and 12 months after chemotherapy initiation, and biannually afterward, until there was evidence of clinical deterioration as defined by radiologic tumor progression, neurological deterioration, or death. Radiologic progression was defined according to the updated response assessment (RANO) criteria for high-grade gliomas encountering also the time from initial chemotherapy as described by Wen et al. [31].

### MR imaging

All MR imaging examinations were performed using the same 1.5T scanner (MAGNETOM Avanto, Siemens Healthcare, Erlangen, Germany) with a 12-channel-array head coil. The imaging protocol included axial FLAIR images (repetition time/echo time (TR/TE) 9000/94 ms, inversion time (TI) 2500 ms, slice thickness 4 mm, intersection gap 10 %, field-of-view 220 × 220 mm) and distortion-corrected T1-weighted images before and after contrast agent administration (TR/TE 275/2.5 ms, slice thickness 4 mm, intersection gap 10 %, field-of-view 230 × 230 mm). All DCE-MRI studies were performed using a 3D fast low angle shot (FLASH) sequence optimized in temporal and spatial resolution in order to provide adequate anatomical coverage, with the following parameters: TR/TE 4/1.4 ms, flip angle 15°, temporal resolution 6 s, base resolution 128, phase resolution 100 %, slice resolution 100 %, 23 slices, slice thickness 4 mm, field-of-view 220 × 220 mm, GRAPPA factor 2, and total acquisition time 5 min. T1 mapping was used to convert signal intensity into gadolinium concentration. The T1 map was calculated from precontrast multiple flip angle images (6°, 12°, and 15°, each acquisition 1 min) with otherwise similar acquisition parameters. Tumors were always situated in the center of the imaging volume; therefore, the difference between nominal and effective flip angles was assumed to be negligible. The gadobutrol administration (0.1 mmol/kg/body weight) was done with a flow rate of 4 ml/s using a power injector, followed by saline flush.

### Image processing and analysis

The conventional and DCE-MR images were consensually reviewed by the same two board-certified neuroradiologists (V.K., S.B.) with experience in central nervous system tumor imaging. The whole tumor volumes were off-line manually delineated in T1 contrast-enhanced images. The DCE-MR images were transferred for post-processing to an off-line workstation running commercially available Olea Sphere™ software, version 2.3 (Olea Medical™, La Ciotat, France). Post-processing included motion correction and rigid-body model registration of precontrast dynamic MR images for conversion of signal intensities into gadolinium concentration. Visual verification and adjustment were used to check and correct any misalignment in the auto-registered images. Briefly, the software analyses transport processes by an open two-compartment model to describe the tissue concentration of the administered contrast agent: a central compartment representing the central blood pool and a peripheral compartment describing the tissue distribution volume (v_e_). The software provides the distribution volume of contrast agent in the tissue: v_e_ (extracellular volume fraction), the tracer exchange between the compartments: K^trans^ based on a modified Tofts–Kermode model, vascular plasma volume fraction: v_p_, and also calculates IAUGC in 60 s [32]. Reverse transfer rate constant k_ep_ is calculated as the ratio of K^trans^ over v_e_. Deconvolution was computed by the standard singular value decomposition method. The arterial input function (AIF) region was selected manually on the right or left internal carotid artery C4 segment to yield the best fitting AIF curve. Internal carotid artery was chosen as basilar or middle cerebral arteries were not equally well visualized for all patients. AIF estimation was model-based and dose-scaled using a bi-exponential function [33, 34]. The calculated DCE-MRI parameters have the following measurement units: K^trans^ in per minute; IAUGC in millimoles per second; k_ep_ in per minute; v_p_ dimensionless; and v_e_ dimensionless [35].

A free, hand-drawn region of interest (ROI) was plotted around the contrast-enhancing tumor in the contrast-enhanced T1-weighted images (paying attention to exclude any large vessels) or around the hyperintense tumor region on FLAIR images, if no contrast enhancement was observed. Tumor delineation and post-processing were performed by a board certified neuroradiologist (S.B.). Other tasks, not requiring qualitative evaluation, were performed by a student with 2 months of software experience (A.U.). The reviewers were blinded to follow up data. The post-processing was repeated in each slice containing tumor tissue, and the parameter values of all ROIs were exported into a spreadsheet. Only the successfully fitted voxels were included in the further analysis. The successful fitting was defined by the following criteria: all perfusion parameters non-negative, 0 < K^trans^ < 4, and 0 < v_e_ < 1. These arbitrary ranges were defined in order to automatically exclude any poorly fitted voxels with unrealistically high or negative values.

### Statistical analysis

The primary outcomes of the study were OS, defined as the number of days from the date of the DCE-MRI examination until death or the last available follow-up date, and PFS, defined as the number of days until progression, verified clinically and by MR imaging, or until the last follow-up date, if no progression or death occurred. One-year PFS and OS were used as cutoffs to stratify patients and evaluate perfusion parameters as prognostic markers.

Data normality was examined by Kolmogorov–Smirnov test. Kurtosis and skewness were calculated for perfusion parameters. Two-sided independent samples Student *t* test was performed for statistical testing of the differences between patient groups. Relations between different perfusion metrics were investigated using Pearson’s product-moment correlations. Survival was analyzed using univariate and multivariate Cox regression analysis models. Receiver operating characteristic (ROC) curve analysis was used to investigate the prognostic value of different parameters for OS and PFS, as well as their diagnostic value for glioma grading and histopathological parameters (e.g., MGMT, IDH). Area under the curve (AUC) was computed, and the optimal cutoffs were calculated by selecting the highest Youden’s J statistic on the ROC curve, thereby maximizing sensitivity and specificity. Kaplan–Meier curve analysis and log rank test were used to detect a difference of survival between patient groups, stratified according to perfusion and clinical parameters. Continuous variables are presented as means with standard deviation, discrete variables as medians with range. Maximum value, 90th percentile, skewness, and kurtosis of perfusion parameters were calculated and are denoted in the text by indices __max_, __90_, __skew_, and __kurt_, respectively. Results were declared statistically significant at the two-sided 5 % comparison-wise significance level (*P* < 0.05). Statistical analysis was performed with SPSS version 22 (SPSS Inc., Chicago, IL, USA).

## Results

### Patient data

**Fig. 1:**
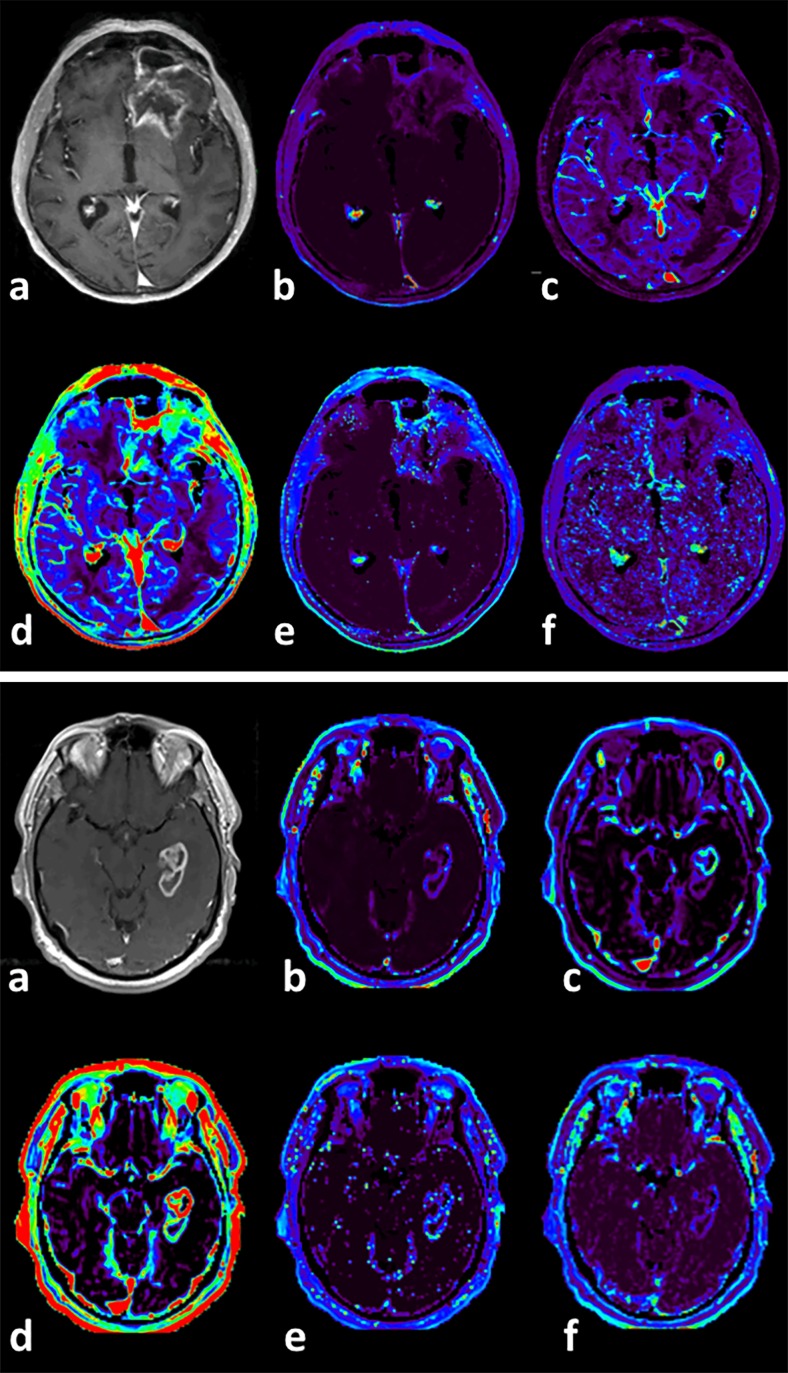
DCE perfusion axial maps of grade III (*above*) and IV (*below*) gliomas. **a** T1 contrast-enhanced; **b** K^trans^; **c** v_p_; **d** IAUGC; **e** v_e_; **f** k_ep_ parameter maps

A total of 69 patients with high-grade gliomas were enrolled in the study. Within this group, 49 (71 %) of the tumors were grade IV (glioblastomas) and 20 (29 %) grade III (anaplastic astrocytomas). Median follow-up of the patients was 424 days (range 23–1686). During the follow-up period, 50 (72 %) patients experienced tumor progression; in 41 (59 %) cases, the progression occurred in less than 1 year after the DCE-MRI scan. Thirty-two (46 %) patients had died by the end of the study. Furthermore, 26 % of the patients were positive for IDH mutation, 39 % had methylated MGMT, and median MIB-1 was 20 % (range 0–80 %). Twelve patients (17 %) received radiation therapy only, 3 (4 %) chemotherapy only, and 54 (78 %) combined chemotherapy and radiation therapy. The 22 (32 %) non-enhancing gliomas included 16 anaplastic astrocytomas (80 % of all grade III gliomas) and 6 GBMs (12 % of all GBMs). Examples of grade III and IV glioma DCE perfusion maps are shown in Fig. 1.

### Perfusion parameter descriptive statistics

After excluding poorly fitted voxels from the histogram data, 2092 (median, range 88–17,590) voxels were analyzed per patient. The mean, median, standard deviation, and range of the maximum, 90th percentile, skewness, and kurtosis values of perfusion parameters of individual patients’ histograms are summarized in Tables 1 and 2.

**Table 1:** Summary of patients’ perfusion parameters—maximum and 90th percentile

	K^trans^_max_	K^trans^_90_	v_p_max_	v_p_90_	IAUGC_max_	IAUGC_90_	v_e_max_	v_e_90_	k_ep_max_	k_ep_90_
Mean ± SD	0.47 ± 0.58	0.11 ± 0.16	0.36 ± 0.32	0.12 ± 0.12	46,974.33 ± 47,288.51	20,249.26 ± 24,615.94	0.87 ± 0.31	0.32 ± 0.35	1.94 ± 1.75	0.65
Grade III	0.39 ± 0.56	0.06 ± 0.16	0.32 ± 0.27	0.11 ± 0.09	29,221.01 ± 24,421.54	11,695.90 ± 14,174.21	0.78 ± 0.40	0.07 ± 0.10	3.06 ± 2.00	1.10 ± 0.98
Grade IV	0.50 ± 0.59	0.13 ± 0.15	0.38 ± 0.34	0.13 ± 0.13	54,220.58 ± 52,411.33	23,740.42 ± 27,128.56	0.91 ± 0.27	0.42 ± 0.36	1.48 ± 1.42	0.46 ± 0.58
*P* value	0.503	0.096	0.420	0.429	0.009^a^	0.019^a^	0.127	0.000^a^	0.003^a^	0.001^a^
Median	0.30	0.06	0.24	0.08	31,176.98	11,570.06	1.00	0.18	1.49	0.44
Minimum	0.00	0.00	0.00	0.00	9.04	2.00	0.00	0.00	0.00	0.00
Maximum	3.17	1.00	1.00	0.41	244,796.72	148,448.71	1.00	1.00	6.80	3.68

^a^Significant difference

**Table 2:** Summary of patients’ perfusion parameters—skewness and kurtosis

	K^trans^_skew_	K^trans^_kurt_	v_p_skew_	v_p_kurt_	IAUGC_skew_	IAUGC_kurt_	v_e_skew_	v_e_kurt_	k_ep_skew_	k_ep_kurt_
Mean ± SD	3.76 ± 4.84	44.35 ± 140.76	2.35 ± 2.26	13.45 ± 37.13	2.19 ± 2.68	13.73 ± 46.49	5.38 ± 6.11	79.94 ± 149.08	4.04 ± 6.98	67.42 ± 283.05
Grade III	6.94 ± 7.05	112.72 ± 242.31	2.10 ± 1.37	8.40 ± 8.56	2.32 ± 1.00	8.51 ± 6.75	10.38 ± 6.91	194.28 ± 194.51	4.26 ± 6.28	59.95 ± 159.9
Grade IV	2.46 ± 2.75	16.44 ± 45.66	2.46 ± 2.54	15.51 ± 43.69	2.14 ± 3.15	16.14 ± 56.84	3.34 ± 4.40	33.28 ± 94.03	3.96 ± 7.30	77.06 ± 336.9
*P* value	0.012^a^	0.093	0.547	0.475	0.821	0.574	0.000^a^	0.002^a^	0.877	0.838
Median	2.48	8.13	1.66	3.90	1.50	2.66	3.09	11.05	1.98	5.36
Minimum	−0.45	−1.12	0.12	−0.87	−0.03	−1.00	−3.50	−1.27	−1.53	−1.11
Maximum	32.04	1084.60	15.49	271.58	17.39	306.85	24.05	631.13	44.64	2234.52

^a^Significant difference

The average values of perfusion parameters were compared between patients stratified according to 1-year PFS and OS. For patients with less than 1-year PFS, v_e_max_ (0.94 ± 0.19 vs. 0.77 ± 0.42, *P* = 0.044) and v_e_90_ (0.41 ± 0.37 vs. 0.19 ± 0.28, *P* = 0.010) were significantly higher. There was no difference in the means of perfusion parameters for patients with less and more than 1-year OS (*P* > 0.05 for all perfusion parameters).

We also investigated whether perfusion parameters were different according to glioma histology. K^trans^_skew_, IAUGC_90_, IAUGC_max_, k_ep_90_, k_ep_max_, and v_e_90_ were significantly different between grade III and IV gliomas (Tables 1 and 2). However, when only contrast-enhancing tumors were analyzed, no significant differences in K^trans^ and v_e_ kurtosis and skewness could be identified between the grades. No perfusion parameters were significantly different for IDH-positive or methylated MGMT patient groups.

Non-enhancing tumors had significantly higher K^trans^ skewness (*P* = 0.004) and kurtosis (*P* = 0.047), as well as v_e_ skewness (*P* < 0.001) and kurtosis (*P* = 0.001), compared to enhancing tumors. Non-enhancing tumors were also significantly more likely to be grade III than grade IV on chi-square test (*P* < 0.001).

### Univariate and multivariate survival analysis

DCE perfusion, histology, and clinical parameters were analyzed as prognostic markers of PFS and OS with Cox proportional hazards model. Parameters that were significant in the univariate analysis and their associated hazard ratios are presented in Table 3.

**Table 3:** Univariate analysis of significant prognostic perfusion and clinical parameters

	OS			PFS		
Parameter	Hazard ratio (HR)	95 % confidence interval	*P* value	Hazard ratio (HR)	95 % confidence interval	*P* value
Age	1.06	1.02–1.09	0.001	1.05	1.03–1.08	0.000
Glioma grade	7.15	1.71–29.97	0.007	3.45	1.59–7.46	0.002
KPS	0.98	0.96–0.99	0.007	0.98	0.97–1.00	0.010
IDH	0.16	0.04–0.69	0.014	0.28	0.12–0.68	0.005
v_p_skew_	1.18	1.03–1.35	0.020	0.83	0.47–1.45	0.512
v_p_kurt_	1.01	1.00–1.02	0.006	0.86	0.49–1.50	0.599
v_e_90_	3.05	1.18–7.89	0.021	2.69	1.22–5.94	0.014
v_e_skew_	0.93	0.86–1.01	0.070	0.94	0.88–1.00	0.035
k_ep_90_	0.46	0.21–0.98	0.044	0.79	0.51–1.21	0.277

### OS analysis

In the univariate analysis of OS, v_e_90_ and k_ep_90_ were the most important significant prognostic factors (higher v_e_ and lower k_ep_ were associated with poorer survival) (Table 3). v_p_skew_ and v_p_kurt_ were also prognostic, however, without significant hazard ratio (HR) values. Higher v_e_skew_ had a small negative non-significant impact on OS (HR 0.93, 95 % CI 0.86–1.01; *P* = 0.070). Among the clinical and histological parameters, age (>55 years), WHO tumor grade (GBM), Karnofsky performance score (KPS) (below 90 %), and absence of IDH1 or IDH1 mutation status were significant negative predictors of OS. On the contrary, MIB-1 and MGMT were not significant. Multivariate analyses were run for perfusion and histological–clinical parameters. Glioma grade and patient age were not included in the models, as they rendered all other parameters insignificant. In the multivariate model including histological and clinical metrics, only KPS (<90 %) had a small negative impact on OS (HR 0.96, 95 % CI 0.94–0.99; *P* = 0.012). IDH, MIB-1, and MGMT status as well as the perfusion parameters did not reach statistical significance.

### PFS analysis

One-year PFS univariate analysis yielded HRs comparable to those of OS. However, v_p_skew_, v_p_kurt_, and k_ep_90_ did not reach significant level, while v_e_skew_ became significant. Therefore, v_e_90_ and v_e_skew_ were the only significant DCE perfusion predictors for 1-year PFS (Table 3). In a multivariate model including the significant predictors of univariate analysis—KPS, IDH, v_e_90_, and v_e_skew_—positive IDH (HR 0.27, 95 % CI 0.10–0.73; *P* = 0.010) and v_e_90_ (HR 3.85, 95 % CL 1.35–10.95; *P* = 0.012) remained significant predictors of PFS.

### Kaplan–Meier survival analysis

**Fig. 2:**
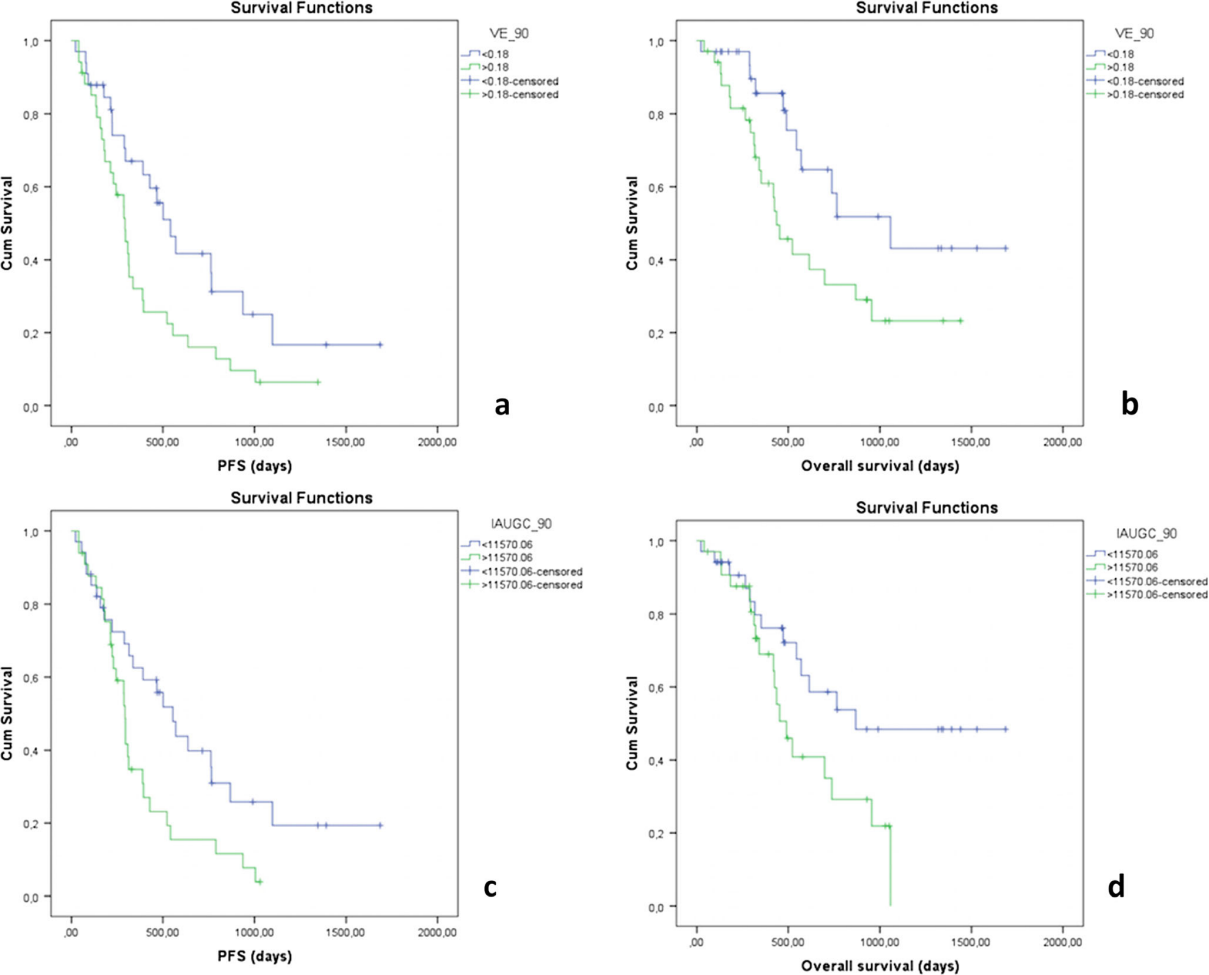
**a**, **b** Kaplan–Meier curves of v_e_90_ for PFS and OS; **c**, **d** Kaplan–Meier curves of IAUGC_90_ for PFS and OS

For the Kaplan–Meier curve analysis, patients were stratified into groups below and above the median of the parameter analyzed (median values are presented in Tables 1 and 2). Perfusion parameters predicting significantly shorter PFS were high v_e_90_, IAUGC_max_, and IAUGC_90_. K^trans^_max_ did not reach statistical significance for PFS prediction *(P =* 0.085), as well as K^trans^_90_ (*P* = 0.108). Significant prognostic factors for OS were v_e_90_ and IAUGC_90_. Significantly different PFS and OS rates are presented in Tables 4 and 5. Kaplan–Meier survival curves for selected parameters are shown in Fig. 2.

**Table 4:** Kaplan–Meier analysis for PFS

		Above median		Below median		
Parameter	Median value	Median survival, days	95 % confidence interval	Median survival, days	95 % confidence interval	*P* value
v_e_90_	0.18	293	226–360	541	398–684	0.021
IAUGC_max_	31,176.98	293	282–304	541	297–790	0.028
IAUGC_90_	11,570.06	293	282–304	554	389–710	0.016

**Table 5:** Kaplan–Meier analysis for OS

		Above median		Below median		
Parameter	Median value	Median survival, days	95 % confidence interval	Median survival, days	95 % confidence interval	*P* value
v_e_90_	0.18	436	315–557	1058	537–1579	0.024
IAUGC_90_	11,570.06	490	369–610	868	612–1124	0.040

### ROC curve analysis

**Fig. 3:**
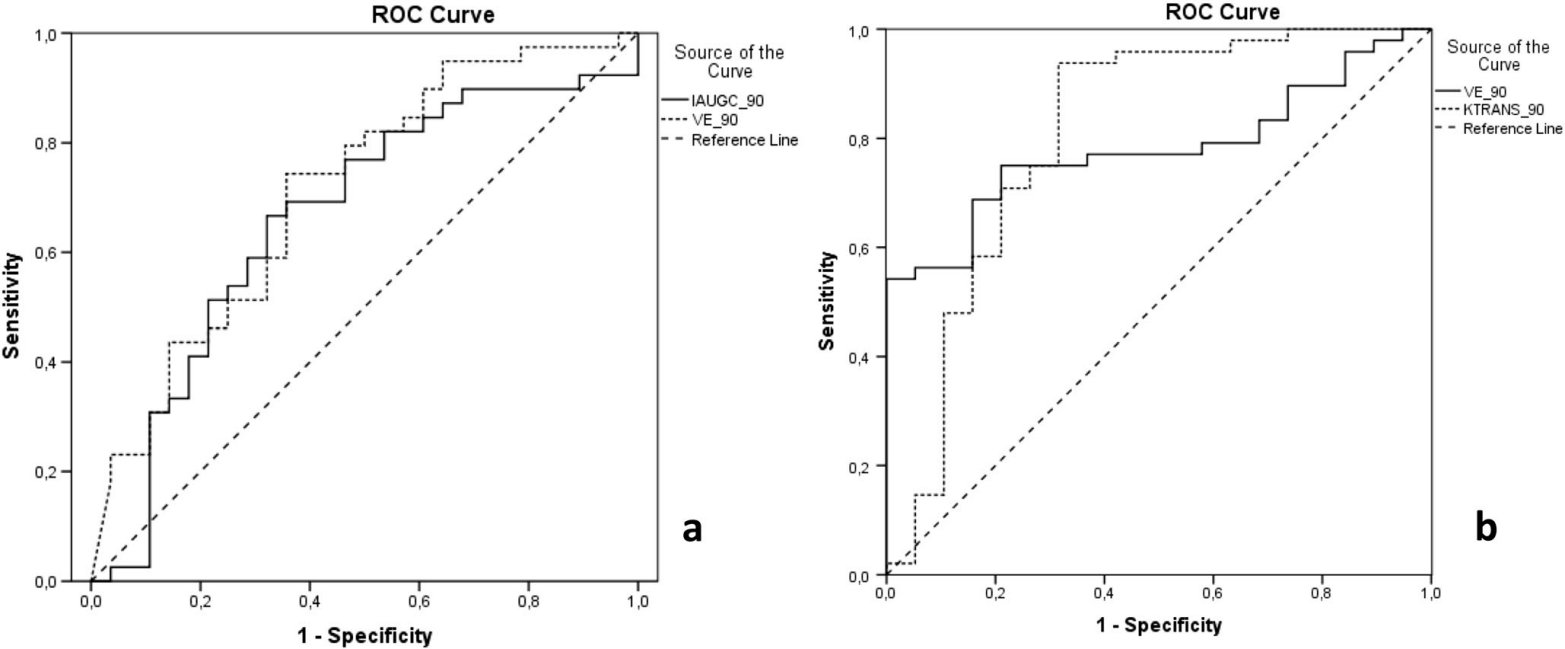
ROC curve analysis. **a** v_e_90_ and IAUGC_90_ for 1-year PFS prognosis; **b** v_e_90_ and K^trans^_90_ for differentiation between anaplastic astrocytomas and GBM

Perfusion parameters achieved weak to moderate prognostic performance for 1-year PFS. The highest AUC was demonstrated by v_e_90_ (AUC = 0.70) and IAUGC_90_ (AUC = 0.66) (Fig. 3a).

ROC curve analysis was also performed to assess DCE perfusion as a tool to differentiate anaplastic astrocytoma and GBM. K^trans^_90_ (AUC = 0.82; optimal cutoff 0.02 min^−1^, sensitivity 0.94, specificity 0.70) and v_e_90_ (AUC = 0.79; optimal cutoff 0.66, sensitivity 0.76, specificity 0.80) showed the best diagnostic value for histological staging (Fig. 3b).

## Discussion

In this study, we found that v_e_ was prognostic for PFS and OS in univariate analysis, in contrast to K^trans^, which had an independent role only in differentiating grade III from IV gliomas. k_ep_ was another predictor of OS, and it was also significantly different between grade III and IV gliomas. Histogram descriptors—v_p_ skewness and kurtosis, and v_e_ skewness—were significant predictors of OS, although the associated hazard ratios were modest.

Few studies have investigated DCE perfusion correlation with high-grade glioma patients’ survival. Nguyen et al. [26] found both higher K^trans^ and v_p_ to be associated with worse OS in a population of mixed grade II–IV astrocytomas, oligoastrocytomas, and oligodendrogliomas, using the hot-spot ROI method. Bonekamp et al. [10] found K^trans^ to be independently associated with worse survival in a sample of 37 GBMs. Compared to DSC-calculated rCBV, K^trans^ was associated with a remarkably higher hazard ratio. Moreover, K^trans^ did not correlate with rCBV closely. In a sample of 18 mixed diagnosis glioma patients with GBMs, oligodendrogliomas, a meningioma, and brain tumors of other histology, Jensen et al. [20] found that v_e_ of peritumoral edema correlates with OS, while blood volume v_b_ correlates with PFS in active tumor regions, and, similar to our study, did not find K^trans^ to be a significant predictor of survival. Analyzing numerous histogram parameters of 61 glioblastomas, Choi et al. [24] found that K^trans^ and v_e_ were associated with worse OS and PFS. Finally, Burth et al. [25] recently found the 90th percentile of K^trans^ both at contrast-enhancing and edema parts of glioblastoma not to have significant prognostic value (interestingly, only the 90th percentile of rCBV of the contrast-enhancing part was a significant predictor of PFS from all imaging biomarkers investigated, leaving apparent diffusion coefficient (ADC) as insignificant as well).

Our study differed from the aforementioned ones in a number of aspects. We have included only astrocytomas (anaplastic and GBM) to have a more homogeneous sample, as oligodendrogliomas are known to have different perfusion characteristics, which could bias DCE perfusion analysis [36, 37]. We used histogram analysis instead of the hot-spot ROI maximum value method. Histogram analysis has been shown to be more reproducible [38] and also enables to calculate tumor voxel statistical distribution parameters: skewness and kurtosis. Until now, correlation of these DCE perfusion parameters and survival has been investigated minimally.

Lack of research on the other than K^trans^ parameters could be because only K^trans^ and IAUGC were originally recommended as the primary end points for perfusion studies [39]. However, even the correlation between K^trans^ and survival has not been firmly established as well. In an early study on the topic, Mills et al. [27] surprisingly found higher K^trans^ to result in longer survival. This trend is indeed observed in some non-brain tumors: metastatic renal cell carcinoma and possibly hepatocellular carcinoma treated with VEGF inhibitors [40], as well as head and neck squamous cell carcinoma treated with chemoradiation [41]. Higher K^trans^ is associated with prolonged OS presumably by enhanced drug delivery through more permeable capillaries in the tumor. However, subsequent glioma studies have shown the opposite trend. Gliomas with lower vascular permeability (K^trans^) and a pronounced decrease of it on the course of treatment are associated with longer OS after chemotherapy [21] and radiation therapy [23, 42], although contradicting results have been published as well [43]. Vascular permeability, measured as K^trans^, could indirectly reflect oxygenation status and thereby predict the response to radiotherapy. It is also possible that lower permeability profile is prognostic rather than predictive and could be associated with better survival regardless of the treatment.

In our study, the most consistent predictor of survival, significantly higher in patients with less than 1-year PFS, and also a significant prognostic factor for PFS and OS in univariate analysis as well as in Kaplan–Meier analysis, was v_e_. On multivariate analysis, only v_e_ remained an independent prognostic factor for PFS. v_e_ represents the extracellular volume fraction or the leakage space, where the contrast agent accumulates after escaping from the intravascular compartment [44], and is thought to reflect the extravascular architecture of the tumor [39]. In preclinical studies, v_e_ has been found to correspond well to the histological extracellular volume [45], coincide with histological necrosis and apoptosis [46], and correlate negatively with cellularity in glioma models [47]. Although extracellular volume could be expected to correlate with ADC, as both parameters reflect cellular density, direct correlation between v_e_ and ADC was not found [11]. This suggests that current understanding of the exact physiologic meaning of v_e_ might be incomplete. v_e_ should not be seen strictly as space between cells and microvasculature/macrovasculature but rather as a parameter in the applied model of the tracer kinetics, therefore subject to some constrains. The accurate estimation of v_e_ is dependent on the flow and permeability of the tumor vasculature [48]. As the tracer traverses the vasculature, a large interstitial space v_e_ would require a substantial amount of tracer to be extracted from the vascular space for the interstitial space to reach a steady-state concentration. For instance, if the flow is very high or v_p_ is large relative to v_e_, steady state could be approached relatively quickly in about 5–6 min, as the tracer traverses through the tissue largely within the vascular space. In this case, v_e_ estimation is reliable. However, tracer extraction (influx) into the interstitial space depends on the capillary permeability. If PS (permeability surface-area product) is low, tracer influx is slow and the corresponding contrast agent concentration curve in tissue approaches its steady-state concentration slowly. In that case, 5–6-min acquisition underestimates the real v_e_. If PS is high, the tracer influx is high and in the given acquisition time (5 min in our study) v_e_ estimation reflects partly this pathophysiology. Thus, the higher v_e_ reflects fast tracer kinetics and is probably due to high permeability, a known biomarker of malignancy itself.

The mechanism on how v_e_ is associated with tumor aggressiveness is still investigated. A recent study by Mills et al. [49] provided some clues: although no relation was found between v_e_ and cell density, v_e_ was positively correlated with mitotic activity. The results are counterintuitive: high mitotic activity is expected in tissue areas with higher cellular density, and not higher extracellular volume fraction. According to Mills et al., this suggests that v_e_ does not reflect extracellular volume directly, possibly due to model flaws. Alternatively, high mitotic activity might not be associated with high cellular volume when malignant cells are very small or exhibit aberrant response to tumoral growth factors and contact inhibition. v_e_ might also reflect microscopic necrotic regions or chaotic tissue architecture, which are also dominant features of higher-grade gliomas. In any case, v_e_ has been found to be higher in HGG than in low-grade gliomas in other studies [12, 14, 15, 50] and was higher in enhancing tumors undergoing progression during concurrent radiation and chemotherapy [51]. Conversely, high v_e_ of peritumoral edema was correlated with better OS [20]. This effect was hypothesized to be predictive: early blood–brain barrier disruption at the outskirt of tumor may facilitate immune response and delivery of chemotherapy agents.

Skewness of v_e_ was also a significant predictor of PFS, but its clinical importance in the model was negligible (the associated HR was 0.94). Although this suggests that higher v_e_ skewness might have a positive effect for PFS, the trend could easily change in a larger cohort. High positive skewness means that there are asymmetric outliers to the far right of the histogram, whereas high kurtosis indicates a sharper histogram peak around the mean values (such distribution could also be called leptokurtic) [52]. Higher skewness—resulting primarily from the contrasting vascular properties of the hypoxic/necrotic core and highly vascularized rim—reflects greater tumor heterogeneity and aggressiveness, thus possibly relating to worse prognosis. Persistent high skewness with a long tail to the right has been linked to worse treatment response in various non-brain tumors [52].

Higher IAUGC in our study was associated with worse PFS and OS on Kaplan–Meier analysis and was significantly higher in grade IV than in grade III gliomas. IAUGC is a simple and robust metric of perfusion, as it does not require a model or curve fitting. It is more resistant to poor fitting at extremely well or poorly vascularized regions, as well as during minute physiological fluctuations. IAUGC is calculated from the area under the contrast agent early uptake curve until a specified time (usually 60 s) [39]. Similar to K^trans^, IAUGC could identify the highly vascularized and permeable tumor volume [39]. Indeed, IAUGC significantly correlated with K^trans^ in our study. On the downside, compared with K^trans^, physiological interpretation of IAUGC is limited.

In our study, v_p_ was not a prognostic factor of OS or PFS. v_p_ is the proportion of blood plasma volume per unit volume of tissue [53]. To this end, it reflects vascularization of tumor and could relate to more aggressive course, similar to rCBV of DSC perfusion. Nguyen et al. [26] have previously found v_p_ to have a prognostic OS value for glioma patients in univariate, but not multivariate analysis, but it was not a significant prognostic factor in other studies [24]. We have found that only skewness and kurtosis of v_p_ were significant negative predictors of OS in univariate analysis, even though the associated HRs were minimal (1.12 for v_p_skew_ and especially 1.01 for v_p_kurt_). As discussed before, high skewness signals higher heterogeneity and outliers with unusually high values, which are attributes of a more aggressive glioma. High kurtosis reflects a more peaked distribution of values, but is argued to be a less precise and a more difficult to interpret parameter.

k_ep_ in our study was also a significant positive prognostic factor for OS in univariate analysis. k_ep_ represents the reverse transfer of contrast material from the extravascular into the intravascular compartment. When the extravascular compartment (v_e_) is large, the transferred contrast agent tends to accumulate there, which results in delayed reverse transfer and decreased rate. Accordingly, higher k_ep_ could be expected to have an opposite prognostic value than v_e_. It was indeed observed in our study.

A number of perfusion parameters were significantly different for grade III vs. IV gliomas (Tables 1 and 2), with the best discrimination achieved by K^trans^_90_ and v_e_90_. These two parameters have already been shown to be different between grade III and IV [12, 16], between HGGs and LGGs [12, 14–16, 50], and between grade II and III oligodendrogliomas [54]. In our study, k_ep_90_ was also significantly lower in grade IV gliomas. We have also investigated kurtosis and skewness of K^trans^, v_p_, v_e_, k_ep_, and IAUGC as predictors of glioma grade. In a study of grade II and III astrocytomas and oligodendrogliomas, Falk et al. [55] found that K^trans^ skewness was higher for grade III gliomas. The difference was in fact below the accepted statistical significance (*P* = 0.07), but it was more significant than any other DCE parameter. In our study, grade III and IV gliomas were compared instead of grade II and III, and K^trans^_skew_ for grade IV was unexpectedly lower than that for grade III. We have not found any study comparing K^trans^ skewness between grade III and IV gliomas, but we believe that our results may be biased due to the uneven distribution of non-enhancing tumors in grades III and IV. Significantly, more non-enhancing tumors were grade III (*P* < 0.001) and had higher K^trans^ skewness and kurtosis than grade IV (*P <* 0.05), possibly due to ROI including tumor and edema together, thus increasing heterogeneity. When blood–brain barrier-disrupted (contrast-enhancing) gliomas of grade III and IV were side-by-side compared, no significant differences of skewness and kurtosis could be found (*P* > 0.05). This stresses the importance of precise tumor segmentation and separate analysis of contrast-enhancing fraction, necrosis, and edema for heterogeneity analysis.

IDH mutation status was a significant prognostic factor of PFS and OS on univariate analysis, while KPS was prognostic for OS. IDH mutation is a well-known positive prognostic factor for the malignant transformation and OS of low-grade gliomas [56, 57]. Previously, some DSC perfusion metrics in a study of 52 HGG patients has been shown to be predictive of IDH mutation—IDH mutation-positive tumors had a more heterogeneous microenvironment [58]. We did not find any correlates of DCE perfusion and IDH mutation or MGMT methylation status, but this does not preclude discoveries in larger cohorts.

Our study is subject to a few limitations. Our sample size was relatively small (*N* = 69), which could explain why other than v_e_ perfusion parameters were not significant prognostic factors in multivariate analysis. We have tried to make the post-processing as standard as possible, but variation could arise when tumor area was delineated by hand. We could not evaluate interobserver variability formally as tumor delineation was performed only once. Another limitation was that we did not compare the prognostic value of DCE perfusion parameters with more established imaging biomarkers, such as rCBV or ADC. However, the primary goal of this study was to perform DCE perfusion parameter histogram analysis and investigate their direct relation with PFS and OS. To this end, we have succeeded in finding the most promising of them. Calibration of DCE perfusion prognostic value to other MRI biomarkers and subsequent optimal integration into standard imaging and evaluation protocol is left for future studies.

## Conclusion

v_e_ was the most consistent predictor of PFS and OS in high-grade glioma patients. v_e_90_ and v_p_skew_ were negative, and k_ep_90_ was a positive prognostic factor for OS in univariate analysis, while v_e_90_ was also a negative prognostic factor for PFS. K^trans^_90_ and v_e_90_ showed the best performance differentiating grade III and IV gliomas on ROC curve analysis.
